# Pathogenicity and immune modulation of porcine circovirus 3

**DOI:** 10.3389/fvets.2023.1280177

**Published:** 2023-11-27

**Authors:** Dengjin Chen, Lei Zhang, Shengkui Xu

**Affiliations:** ^1^Key Laboratory of Veterinary Biological Products and Chemical Drugs, Ministry of Agriculture and Rural Affairs, Engineering and Technology Research Center for Beijing Veterinary Peptide Vaccine Design and Preparation, Zhongmu Institutes of China Animal Husbandry Industry Co., Ltd., Beijing, China; ^2^Beijing Key Laboratory of Traditional Chinese Veterinary Medicine, College of Animal Science and Technology, Beijing University of Agriculture, Beijing, China

**Keywords:** porcine circovirus 3 (PCV3), porcine circovirus associated disease (PCVAD), advance, pathogenicity, immune modulation

## Abstract

Porcine circoviruses (PCVs) are members of the genus *Circovirus* of the family *Circoviridae*, and four species of PCVs have been discovered and named PCV1–PCV4, respectively. With the first report of PCV3 in America in 2016, the pathogenic variant was found to be associated with various clinical features, called porcine circovirus associated disease (PCVAD), including multisystemic inflammation, porcine dermatitis and nephropathy syndrome (PDNS), reproductive disorders, respiratory or digestive disorders. Increasing experimental data have shown that PCV3 is widespread around the world, but the failure of virus isolation and propagation has put obstacles in the way of PCV3 research. Moreover, a large number of reports demonstrate that PCV3 usually co-infects with other pathogens in pigs. Thus, whether PCV3 alone causes clinical manifestations needs to be fully discussed. In addition, the host cell immune response was activated during PCV3 infection, and PCV3-encoded proteins may regulate immune responses to facilitate its replication. An in-depth understanding of PCV3 pathogenesis and immune regulation strategies is critical for PCVAD prevention. In this review, the advances in pathogenicity and innate immune modulation of PCV3 were summarized, which could deepen the understanding of this virus and PCV3-related diseases.

## 1 Introduction

Porcine circoviruses (PCVs), the smallest known DNA viruses in mammals, are members of the genus *Circovirus* of the family *Circoviridae*. At least four species of PCVs have been discovered in pigs and named PCV1 to PCV4, respectively, based on the genome homology analysis. In the 1970s, PCV1 was first found in PK-15 cells and then proved non-pathogenic in subsequent studies (1, 2). Subsequently, a variant strain of PCV2 was found in North America and Europe and thought to be responsible for multiple clinical presentations, such as post-weaning multisystemic wasting syndrome (3–5). Therefore, extensive studies were carried out, and great progress was made on viral pathogenesis and immune regulation mechanisms in the following 20 years. In 2016, a new virus, causing similar symptoms to PCV2, was reported and named PCV3 (6). In addition, PCV4 was recently discovered in 2019 (7), and there is only limited information about the pathogenesis and clinical implications of PCV4.

PCV3 was initially detected in the United States in pigs suffering from porcine dermatitis and nephropathy syndrome (PDNS) using high-throughput sequencing technology (6). However, numerous retrospective studies suggest that the pathogen has been circulating among pigs for a long time in Asia, Europe, South America, and North America (8–14). Notably, most of the PDNS pigs are PCV3-positive but not PCV2, and the PDNS-like disease in piglets was successfully reproduced based on an infectious PCV3 DNA clone (15), indicating PCV3 is sufficient to cause PDNS solely, and much investigation should be done to unveil its biology and pathogenesis. Moreover, PCV3 was highly prevalent in wild boars (16–21). Thus, the wild populations may be an important reservoir and could represent a concrete risk of spreading to domestic populations.

In general, all PCVs share a similar morphology, consisting of a close circular single-stranded DNA genome (22). The genome consisted from about 1760 nucleotides (nt), 1767 nt, and 2000 nt in PCV1, PCV2, and PCV3 (23), respectively. In addition, the sequence of PCV3 was only 48% homologous with PCV2, although they share similar genome morphologies (24). Further phylogenetic analysis also demonstrated the low similarity between PCV3 and other PCVs, such as 45.5 and 43.2% for PCV1 and PCV4, respectively (25). Recent studies reported that the novel PCV3 might originate from bats due to its high homology to some bat circoviruses at both nucleotide and amino acid levels (26, 27).

PCVs consist of three major open reading frames (ORFs) arranged in the strands of the replicative form (28). ORF1 is located on the positive strand and identified as the most conserved region of the circovirus genome (29), encoding for Rep and Rep′ proteins involved in virus replication initiation (30); ORF2 is located on the negative DNA viral strand and encodes the only structural protein (Cap), which is the most immunogenic viral protein and possesses multiple functions (31); ORF3 is oriented in the negative strand, which encodes for a non-structural protein regulating host cell apoptosis in PCV1 and PCV2 (32, 33), while its function remains unknown in PCV3 (6, 34). Traditionally, PCV3 is divided into three main clades (PCV3a, PCV3b, and PCV3c) based on the amino acid variations (35, 36), and PCV3a could be further classified into subclades, including PCV3a-1, PCV3a-2, and one intermediate clade (PCV3a-IM) (26, 27, 35, 36).

Since the first report of PCV3, it has been widely detected in the lungs, heart, kidneys, and other organs or tissues (15) and has caused a variety of clinical manifestations (37). The current research proves that various confounding factors affect the clinical manifestations and pathological changes of diseased pigs (38). PCV3 can replicate in almost all tissues, especially immune cells, where it causes targeted cell damage such as cell apoptosis and immune suppression. In addition, PCV3 infection could significantly upregulate pro-inflammatory cytokines and cause multisystemic inflammation in piglets and sows (15). Therefore, the interplay between PCV3 infection and immune responses is critical to its pathogenicity. In this review, we will comb and organize the pathogenicity and immune modulation mechanisms of PCV3.

## 2 PCV3 pathogenicity and its associated diseases

**Figure 1:**
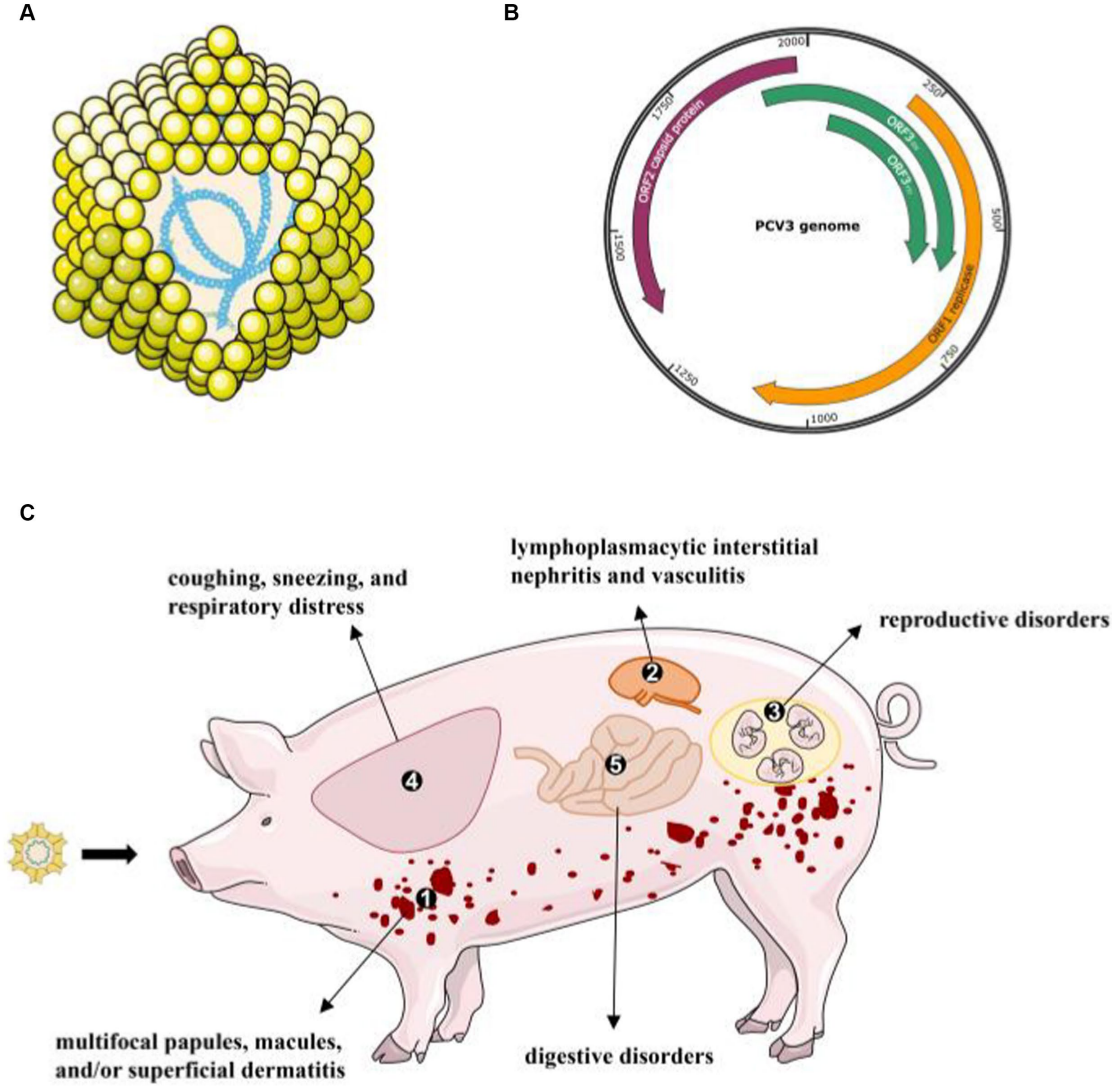
The virion structure of PCV3 and its clinical diseases or signs **(A)** PCV3 has a single-stranded, circular DNA genome, and the capsid consists of 12 pentagonal, trumpet-shaped pentamers. **(B)** The PCV3 genome is about 2000-nt in length and contains three major ORFs: ORF1, ORF2, and ORF3 (45). **(C)** A variety of clinical and pathological symptoms are associated with PCV3 infection: ① skin: multifocal papules, macules, and/or superficial dermatitis; ② kidney: lymphoplasmacytic interstitial nephritis and vasculitis; ③ reproductive disorders: abortion, stillbirth, and mummified fetuses; ④ lung: coughing, sneezing, and respiratory distress associated with lymphoplasmacytic interstitial pneumonia; ⑤ digestive disorders.

A series of clinical symptoms caused by PCVs are collectively known as porcine circovirus-associated diseases (PCVAD). In the past 20 years, PCV2 has been regarded as the main cause of PCVAD. PCV2 mainly proliferates in lymph nodes and induces immune cell apoptosis to decrease the immunity of infected pigs. Exciting progress has also been made in PCV3 pathogenicity. Jiang et al. successfully reproduced PDNS-like disease based on PCV3 alone or PCV3 in combination with an immune stimulator in animals (15). It is clear from the evidence that PCV3 infection leads to a variety of clinical and pathological symptoms, including PDNS, reproductive disorders, systemic inflammatory diseases (39), respiratory disorders (34, 40, 41), diarrhea (40, 42), and central nervous system signs (43, 44). Here, we describe the recent advances in the most consistent clinical signs associated with PCV3 infection (Figure 1).

### 2.1 Multisystemic inflammation

PCV3 was initially discovered in sows and aborted fetuses that had PDNS-like clinical symptoms and reproductive dysfunction (6). PDNS is mainly harmful to nursery and finishing pigs, with the occasional onset of piglets. The disease has a low mortality rate but a high incidence and a long course and is clinically characterized by irregular erythema of multifocal papules and superficial dermatitis. Currently, studies have shown that PDNS-like disease is usually the most severe form of the disease that is present in pigs that are naturally and experimentally infected with PCV3 (15, 46, 47). Typical PDNS lesions such as necrotizing vasculitis and glomerulonephritis could be observed in sows (48, 49). PCV3-infected sows and aborted fetuses showed multiorgan inflammation (39) and multisystemic vasculitis with a prominent skin and kidney tropism caused by type III hypersensitivity reactions (50). A wide range of vasculitis, from local to systemic, has been reported in PCV3-infected pigs (6, 15, 39, 44, 46, 51). Furthermore, vasculitis has also been found in the heart, kidney, and intestinal tissues of PCV3-inoculated pigs (51, 52). In addition, hepatic pathology, such as granulomatous lymphadenitis, has been demonstrated in the PCV3 challenge experiment (15, 53). Moreover, a high level of chemokines and pro-inflammatory mediators was detected in the PCV3-infected pigs (15), and it may be responsible for multisystemic inflammation, and host immune responses, and the observed lesions described above.

### 2.2 Reproductive disorders

Although PCV3 did not always lead to symptomatic signs in sows, the virus was widely colonized in gilts, weak-born piglets, and in the fetuses and placenta (51, 54–58), PCV3 could also be transmitted vertically through the colostrum, semen, and placenta (59). Reproductive disorder is a disease that reduces the performance of sows, including infertility, abortion, stillbirth, and weakness or neonatal malformation (60), affecting the global pig industry’s health development. Existing studies have shown that high levels of PCV3 are detected in pig farms with reproductive failure by qPCR testing (40, 61), and high PCV3 titers could be found in the aborted fetuses (46, 57), indicating that PCV3 infection could directly affect the fetuses (44). Although PCV3 can be detected in sows with or without reproductive disorders, the positive rates of PCV3 are much higher in reproductive failure sows than those in healthy sows via qPCR assays (61). In addition, high PCV3 viral loads were detected using qPCR in the various tissues, including the fetal heart, thymus, lymph nodes, and placenta, suggesting additional tissue tropism of PCV3 (62). Interestingly, the virus could also be detected in multiple tissues of mummies or stillborn fetuses, such as the trophoblast cells, the placenta, and the umbilical cord (21, 44, 62, 63). Thus, infection of the fetuses was thought to contribute to the reproductive failure induced by PCV3. However, the mechanism underlying PCV3-induced reproductive failure remains unclear and requires further study.

### 2.3 Respiratory or digestive disorders

Pigs, especially weaned piglets, infected with PCV3 typically exhibit various clinical symptoms, including respiratory diseases and digestive disorders (6, 35, 40, 64). However, the association between PCV3 circulating status and clinical respiratory manifestation is not well understood. Zhai et al. systemically analyzed and found that the PCV3 positive rate was positively correlated with respiratory symptoms (40). Similarly, a much higher percentage of diarrheal-weaned pigs were PCV3 positive than non-diarrheal ones (40). Another report reached a similar conclusion, there was a close association between PCV3 infection and digestive or respiratory diseases (35). Although the results above proved that PCV3 was potentially associated with swine respiratory disease and diarrhea, direct evidence was imperative. To answer this question, specific-pathogen-free (SPF) piglets were intranasally inoculated with a PCV3 virus obtained from the infectious DNA clone by Jiang et al. and they found that the virus infection increased respiratory disease rates (15). A variety of clinical respiratory symptoms were observed in this study, including coughing, sneezing, and respiratory distress.

Previous studies proved that PCV3 was able to replicate in the lungs and that virus infection could activate the innate immune system and trigger the release of inflammatory cytokines (39, 65). Clinical respiratory signs are usually accompanied by interstitial pneumonia and pleuritis, as well as other types of pulmonary lesions (66). Pneumonia will lead to alveolar congestion and edema, and lots of inflammatory secretions in the alveolar cavity will affect oxygen exchange. However, there are some conflicting results. The pigs only demonstrated subclinical infection without any pathological lung changes and showed no evidence of PCV3 replication in the lung tissue (51, 52). Another study showed that rescued PCV3 virus infection in Kunming mice did not affect the tissues or organs of either control or infected groups (67). At the moment, there is not enough information regarding the factors contributing to the lung tropism and pathogenesis of PCV3.

A report shows that there is a tendency for diarrhea in PCV3-positive suckling and weaned piglets (42). Based on immunohistochemistry, it was determined that the PCV3 antigen was present in all organs, including the intestinal tract (65, 68). Consistently, the infected pigs developed diarrhea, with degeneration and necrosis of the small intestinal epithelium in the PDNS-like lesions study (15). The piglets inoculated with PCV3 showed a high level of PCV3-positive cells within their small intestine tissues (69). Experimental infection studies revealed that piglets inoculated with PCV3 showed a series of lesions in the small intestine, including villus and crypt atrophy, eosinophilic and lymphocyte infiltration, mucosal epithelial cells and lymphocyte necrosis, and small numbers of plasma cells (42, 69). Gut microbiota is made up of trillions of bacteria and plays critical roles in modulating host immunity; changes in the microbiota are closely linked to the progression of diseases. Therefore, it may be an effective way to prevent PCV3 infection through the regulation or changes of the gut microbiota.

## 3 Immune modulation of PCV3

**Figure 2:**
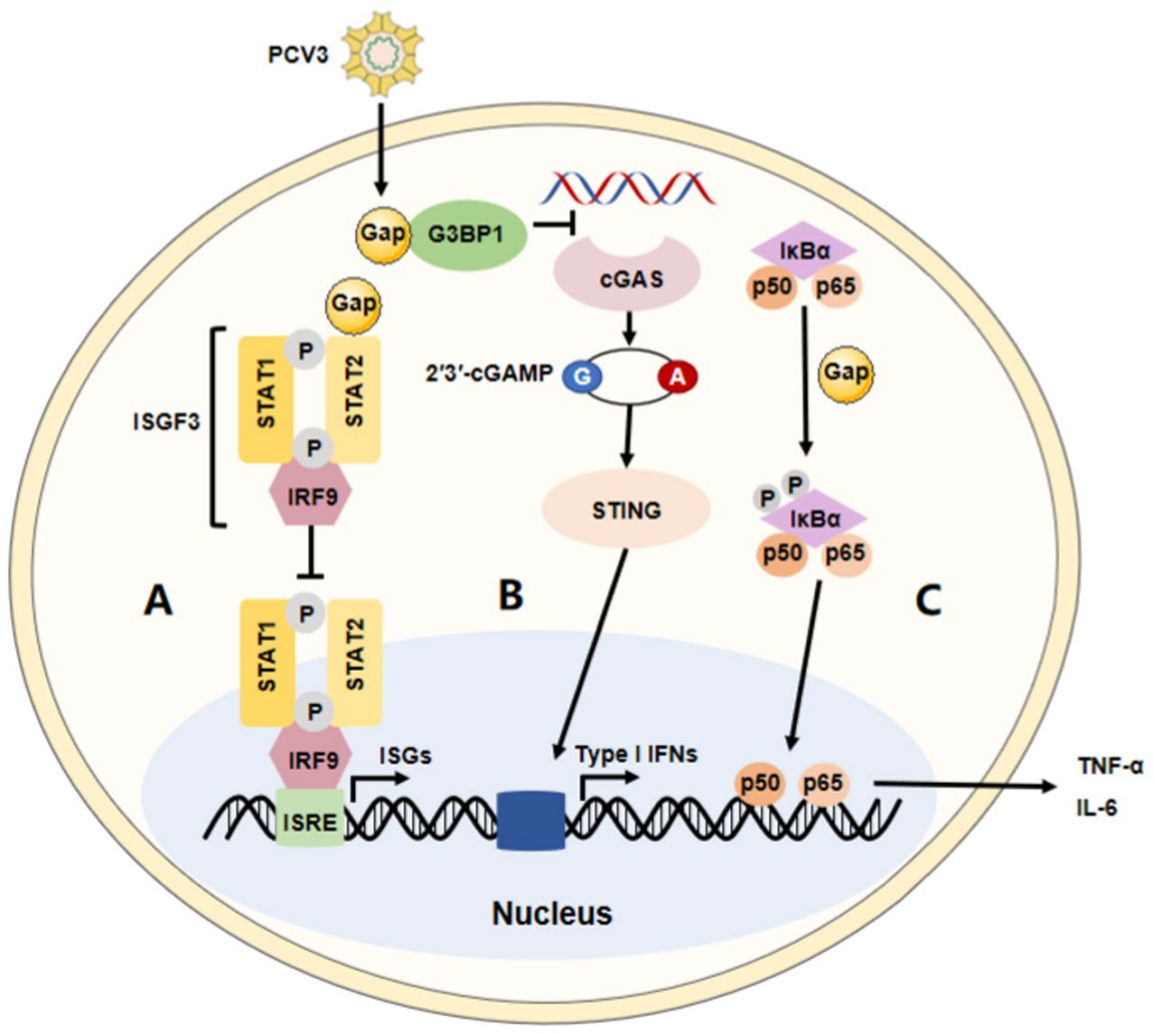
The immune modulation of PCV3. The summary of the major immune modulations of PCV3. **(A)** PCV3 Cap interacts with the transactivation domain of STAT2 and inhibits ISRE promoter activation induced by IRF9-S2C, helping PCV3 to escape type I interferon-meditated host innate immunity. **(B)** PCV3 Cap interacts with G3BP1 to prevent cGAS from recognizing DNA, affecting type I interferon production via the cGAS-STING pathway. **(C)** PCV3 Cap protein induces NF-κB activation and upregulates the expression of pro-inflammatory cytokines.

PCV3 has been widely reported around the world since its discovery and is characterized by typical PDNS lesions. Unfortunately, only one successful PCV3 isolation has been recently reported in cell culture (70), and there may be various unknown factors associated with the disease. Increasing results have shown that PCV3 triggers the host cells’ immune response when invading host cells (Figure 2). High levels of the PCV3 genome could be found in aborted and weak-born piglets, especially in the thymus and lymph nodes (62). Moreover, a few animal detections found that PCV3 exhibits persistent infection (18) or asymptomatic infection (63). Overall, the virus tends to replicate in almost all tissues in pigs, and strongly prefers to replicate in immune cells, and causes targeted cell damage.

### 3.1 Innate immune response

#### 3.1.1 Type I interferon response

A diverse range of defensive strategies are employed by different viruses to battle innate immunity. However, the immune evasion strategies of PCV3 are not fully understood. It has been reported that Cap protein could significantly inhibit the activity of the IFN-stimulated response element (ISRE) by interacting with the transcription (STAT2) transactivation domain (56, 71). Several studies have demonstrated PCV2 not only activates cGAS/STING and RIG-like receptor (RLR) signaling, but it also increases IFN-β expression, which promotes the proliferation of PCV2. Meanwhile, in this process, surprisingly, there seems no appearance to prevent its replication through the NF-κB signaling pathway (72–74). These findings are consistent with those in the studies on PCV3. It has been reported that the PCV3 Cap protein inhibits the type I interferon signaling pathway by inhibiting ISRE promoter activity. Mechanically, Cap interacts with the transactivation domain of STAT2 and binds to ISRE to prevent the binding of STAT2 and ISRE (71). Therefore, PCV3 could evade the host’s innate immunity mediated by IFN. In addition, PCV3 Cap can also interact with G3BP1 and inhibit the induction of type I interferon (75). According to proteomic analysis, PCV3 infection in SPF piglets infected with infectious clones of PCV3 increased several IFN-related factors, such as IFIT3, ISG15, and so on, in lung tissue (47). Another study performed on HEK-293 T cells found that, though it was not observed that PCV3 Cap contributed to the controlled expression of IFN-β, the mRNA level of RIG-I/MDA5 could significantly be increased by Cap protein in the RLRs pathway (76). Therefore, these studies indicate that the regulation of the type I interferon response may be an important strategy for host immune escape by PCV3. Moreover, a recent paper reported that the PCV3 Cap protein is involved in cell autophagy, another mechanism thought to be effectively used by viruses to enhance their replication and persistency (77). Mechanically, the protein induced complete autophagy by inhibiting the phosphorylation of the mammalian target of rapamycin in HEK-293 T cells, including the formation of autophagosomes and autophagic vesicles, as well as the transformation of LC3-I to LC3-II (78). Of course, the ubiquitin-proteasome pathway is also involved in this process. In all, information in this area is necessary to understand the role of pathogenicity and innate immunity during PCV3 infection.

#### 3.1.2 Inflammatory responses

According to previous studies, a close association exists between PCV3 and multisystem inflammation, as well as respiratory disease, diarrhea, myocarditis, encephalitis, periarteritis, and so on (6, 34, 39, 44, 51, 67, 68, 79, 80). Studies have shown that uncontrolled inflammatory responses might contribute to significant damage in PCV3-infected pigs (51, 79). PCV3 infection was demonstrated to have regulatory effects on IL-8 expression by evaluating the innate immune response *in vivo* (52). Significantly, higher levels of pro-inflammatory mediators and chemokines were observed in PCV3-infected piglets (15). In a recent study, PCV3 Cap was shown to be a critical factor in activating NF-κB signaling by upregulating pro-inflammatory mediators such as RIG-I and MDA5 in HEK-293 T cells (76). This may provide a basis for the pathogenesis of PCV3 and the innate immunity of the host. Therefore, the induction of a chronic pro-inflammatory state of disease results in dysregulation of innate immunity in PCV3-infected pigs, which may provide a possible explanation for the subsequent clinical signs. PCV3 Cap protein appears to activate some signaling pathways, but the mechanism is still unclear and needs further investigation.

### 3.2 Cell-mediated immune responses

As a consequence of the innate immune subversion, reduced T-cell response, compromised antigen presentation ability, and the imbalance of immunosuppressive cytokine secretion, the adaptive immune response against PCV2 was severely influenced (81). For example, the proteins of PCV2 (such as Cap and Rep) stimulate antigen-specific IFN-γ secreting cells (82–84). Meanwhile, the infection of PCV2 could directly affect immature thymocytes, such as by inhibiting thymocyte selection, which results in disturbances of helper T cell immunity (85). Even when the immune response to PCV2 is low, pigs still display protective clinical symptoms, indicating that cell-mediated immune responses, specifically Th1-mediated responses, play crucial roles in protective immunity. A study using recombinant PCV3-infected conventional weaning piglets and PBMCs found that they were incapable of responding to mitogen stimulation, but it is unclear whether this effect is permanent or whether energy of lymphocytes can be reversed (15). As a result of proteomic analysis of PCV3-infected pigs, it was found that both SLA-I and II loci in lung tissue were significantly upregulated (47). At the same time, it has been reported that most of the influx of lymphocytes is associated with T-cell populations, regardless of peripheral response to PCV3 (52). However, a more detailed study of PCV3-mediated cell immunity is necessary to delve deeper into the pathogenesis of PCV3.

### 3.3 Immunopathogenesis

All the pathogenic PCV types are widespread and have been detected in both healthy and sick pigs. They have a variety of clinical manifestations and often cause chronic systemic infections. Current research results have suggested that the homeostasis disorder of the immune system may be the key factor leading to the pathogenesis of PCV infection. In PCV2 infection experiments, the virus could interact with components of the immune system, including immune cells, to impair innate and adaptive immunity. Dysregulation of the immune response can lead to a series of serious consequences, including rapid upregulation of pro-inflammatory factors, the formation of immune complexes, decreased antigen presentation ability, and necrosis of lymphocytes and immune cells. For example, several studies have demonstrated that the infections of PCV3 and PCV2 are connected to the immune complexes’ formation (15, 86). Although there are few reports on the immunosuppression of PCV3 to date, considering the similarity of clinical symptoms and pathogenicity between PCV3 and PCV2, we believe that PCV3 may also have strong immunosuppression, especially in the case of co-infection with other viruses, which is also worth exploring in the following study.

## 4 Others

### 4.1 Co-infection

In recent years, multi-pathogen mixed infection or co-infection has become more common in clinical practice, especially with a variety of pig viruses, including PCVs. Co-infection not only leads to more serious diseases than any single virus infection but also has a negative impact on the pig immune system and aggravates the complexity of pig farm diseases. There are reports that the co-infection rates of PCV3 with porcine reproductive and respiratory syndrome virus, classical swine fever virus, pseudorabies virus, porcine epidemic diarrhea virus (PEDV), and porcine parvovirus (PPV) were 36.36, 6.92, 14.53, 27.27, and 74.2% in some pig farms, respectively (87–91), indicating that the co-infection of PCV3 with other pathogens is common in pig farms. Moreover, co-infection of PCV2 and PCV3 was mostly reported in swine farms, although they share a similar genome and belong to the same genus. A survey of serum samples from clinically healthy pigs from major European countries showed a 3% positive rate of PCV2–PCV3 co-infection in fattening pigs (92). And a report found that the PCV2 and PCV3 co-infection rates gradually increased from 3.4% in 2016 to 16.1% in 2018 in the Midwest of the United States (93). In addition, studies have reported that the positive rate of PCV2 and PCV3 co-infection in different regions of China ranges from 6.78 to 19.7% (94, 95). These results indicate that the prevalence of PCV2 and PCV3 co-infection is widespread all over the world and has gradually increased in recent years.

### 4.2 Vaccination

Vaccination has been proven efficacious and successful for the prevention of PCV2 and other viral pathogens. Commercial PCV2 vaccines are mostly inactivated or Cap-based subunit vaccines, which provide favorable protective immunity against other types of PCV2 (68). ORF2 encodes the only structural protein capsid, and the identity between PCV2 and PCV3 was lower. Therefore, the vaccines based on PCV2 may only provide limited protection against PCV3. As expected, there was no correlation between PCV2 vaccination and PCV3 circulation, indicating the cross-protection between PCV2 and PCV3 was poor (96). However, there are no PCV3-based commercial vaccines to prevent PCV3 infection. Therefore, it is urgent to develop efficacious measures like universal antibodies and vaccines to prevent the disease in the future.

## 5 Conclusion and perspective

PCV3 was widespread in the world, including Asia, Europe, and America (8–13), according to retrospective epidemiological studies, although it was first discovered in pigs with PDNS-like clinical signs in 2016 (6). It is generally considered to be pathogenic and associated with various symptoms similar to those of PCV2, including PDNS, reproductive failure, and respiratory diseases. The symptoms described in this paper might not be the major clinical signs of PCV3, and there may be other contributing factors. Thus, the impact of PCV3 is still controversial to some extent, and further information is required to understand its potential pathogenicity. PK-15 and porcine testicular cells were susceptible to PCV2 and used for PCV2 isolation and propagation (97), but PCV3 mostly failed to be isolated and propagated in these passage cells (70). Furthermore, only one PCV3 infection model was reported. Therefore, the restrictions above hinder the research into PCV3 pathogenesis. Accumulating data indicated that PCV3 is often co-infected with PCV2, PPV, PEDV, and other pathogens, which not only lead to the severity of the disease, but also become a serious threat to the healthy development of the pig industry system (56, 58, 98). Moreover, PCV3 infection has a low mortality rate but a high morbidity and a long course of disease, which may be a great threat to the pork industry. Thus, it is imperative to further systematically evaluate and investigate the co-infection prevalence and pathogenicity of PCV3 with other pathogens and to develop an effective vaccine against PCV3.

## Author contributions

DC: Data curation, Investigation, Methodology, Resources, Writing – original draft, Writing – review & editing. LZ: Conceptualization, Writing – review & editing. SX: Funding acquisition, Project administration, Resources, Supervision, Validation, Writing – review & editing.
